# Technology and Social Isolation Among Older Adults

**DOI:** 10.1093/geroni/igaa057.1331

**Published:** 2020-12-16

**Authors:** Allison Dunatchik, Jerry Jacobs

**Affiliations:** University of Pennsylvania, Philadelphia, Pennsylvania, United States

## Abstract

In this paper, we use time-use data from the US (2003-2018) and the UK (2001-2015) to address important questions about recent trends in social isolation and technology use among older adults aged 65 and over. Do new information, communication and robotic technologies contribute to growing social isolation among older adults or to growing opportunities for social connection? We believe that answers to these questions require a consideration of technologies that are already widely in use. We ask whether TV time is social time or whether people spend most of their time watching TV by themselves, whether this has changed over the past 15 years and what the potential implications are for wellbeing among older adults. The results show that TV time is a mixed experience among older adults. In the US and the UK, a substantial proportion of TV time – around half – is spent alone, with those who are not married, those in rented accommodations and those with poorer self-rated health spending more time watching TV alone. Controlling for demographic and household characteristics, TV time alone has increased in recent years, accompanying an increase in time spent alone in general in the UK and an increase in TV time in general in the US. Analysis of moment-to-moment wellbeing shows that individuals report lower positive affect while watching TV alone than while watching with others, suggesting the increase in time spent watching TV alone may have negative implications wellbeing among older adults.

